# Intensity and duration of salinity required to form adaptive response in C_4_ halophyte *Kochia prostrata* (L.) Shrad

**DOI:** 10.3389/fpls.2022.955880

**Published:** 2022-10-07

**Authors:** Elena Shuyskaya, Zulfira Rakhmankulova, Maria Prokofieva, Luizat Saidova, Kristina Toderich, Pavel Voronin

**Affiliations:** ^1^ K.A. Timiryazev Institute of Plant Physiology Russian Academy of Science, Moscow, Russia; ^2^ International Platform for Dryland Research and Education, Tottori University, Tottori City, Japan

**Keywords:** Chenopodiaceae, C_4_ photosynthesis, carbon-concentrating mechanism, photosystems I and II, Rubisco and PEPC, proline, salinity stress, dryland

## Abstract

Plant adaptation to salinity is a highly multifaceted process, harnessing various physiological mechanisms depending on the severity and duration of salt stress. This study focuses on the effects of 4- and 10-day treatments with low (100 mM NaCl) and moderate (200 mM NaCl) salinity on growth, CO_2_/H_2_O gas exchange, stomatal apparatus performance, the efficiency of photosystems I and II (PS I and II), content of key C_4_ photosynthesis enzymes, and the accumulation of Na^+^, K^+^, and proline in shoots of the widespread forage C_4_ halophyte *Kochia prostrata*. Our data show that 4 days of low salinity treatment resulted in a decrease in biomass, intensity of apparent photosynthesis, and cyclic electron transport around PS I. It was accompanied by an increase in transpiration and Rubisco and PEPC contents, while the Na^+^ and proline contents were low in *K. prostrata* shoots. By the 10th day of salinity, Na^+^ and proline have accumulated; PS I function has stabilized, while PS II efficiency has decreased due to the enhanced non-photochemical quenching of chlorophyll fluorescence (NPQ). Thus, under low salinity conditions, Na^+^ accumulated slowly and the imbalance between light and dark reactions of photosynthesis was observed. These processes might be induced by an early sodium signaling wave that affects cellular pH and ion homeostasis, ultimately disturbing photosynthetic electron transport. Another adaptive reaction more “typical” of salt-tolerant species was observed at 200 mM NaCl treatment. It proceeds in two stages. First, during the first 4 days, dry biomass and apparent photosynthesis decrease, whereas stomata sensitivity and dissipation energy during dark respiration increase. In parallel, an active Na^+^ accumulation and a decreased K^+^/Na^+^ ratio take place. Second, by the 10th day, a fully-fledged adaptive response was formed, when growth and apparent photosynthesis stabilized and stomata closed. Decreased dissipation energy, increased WUE, stabilization of Rubisco and PEPC contents, and decreased proline content testify to the completion of the adaptation and stabilization of the physiological state of plants. The obtained results allowed us to conclude that the formation of a full-fledged salt-tolerant response common for halophytes in *K. prostrata* occurs by the 10th day of moderate salinity.

## Introduction

Salinization is a worldwide environmental problem that negatively affects crop yields, posing a threat to global food security. The negative impact of salinity is increasing all the time because of climate change, reinforcing the dependence of agriculture on irrigation and the increasing urbanization rate (Panta et al., 2014; Zelm et al., 2020; Ibrahimova et al., 2021). Given the growing threat of salinity, it is necessary to understand the mechanisms of salt tolerance in plants and find ways to create new salt-tolerant crops (Volkov, 2015; Zelm et al., 2020; Ibrahimova et al., 2021). Saltiness induces both osmotic and toxic stress in plants, which results in decreased growth, developmental changes, metabolic adaptation, and ion sequestration, or exclusion (Munns and Tester, 2008; Munns et al., 2012). Osmotic and ionic toxicity effects have long been thought to be separate in time and space. Such a differential effect suggests that early responses to salt stress arise as a consequence of general osmotic stress, while sodium-specific responses are induced later (Munns and Tester, 2008; Shavrukov, 2013). This concept has been challenged by recent discoveries of rapid salt signaling and rapid sodium-induced root growth response (Galvan-Ampudia et al., 2013; Choi et al., 2014; Zelm et al., 2020). The first plant reactions to salinity occur within seconds or hours of salt stress (Julkowska and Testerink, 2015). Studies of intracellular Ca^2+^ peaks have shown that plants can perceive both the osmotic and ionic components of salt stress (Lamers et al., 2020). Three major early signaling compounds have been identified: glycosylinositolphosphorylceramide (GIPC, sphingolipid); binding to sodium, it can stimulate Ca^2+^ import; 3’,5’-cyclic guanosine monophosphate (cGMP), whose rapid accumulation inhibits sodium influx by deactivating non-selective cation channels (NSCCs); and reactive oxygen species (ROS) (Julkowska and Testerink, 2015; Lamers et al., 2020; Zelm et al., 2020). It was shown that the Salt Overly Sensitive (SOS) signaling pathway plays a key role in decoding Ca^2+^ signals and maintaining ionic homeostasis. Additionally, mitogenactivated protein kinase (MAPK) cascades mediate ionic, osmotic, and ROS homeostasis (Yang and Guo, 2018; Lamers et al., 2020; Zelm et al., 2020; Chen et al., 2021). However, there is still much that is unclear about early cellular responses to salinity and sodium import, since such studies are fragmentary and insufficient (Zelm et al., 2020).

One of the early responses of the whole plant to salinity is a decrease in photosynthesis. The effect of salinity on photosynthesis is usually associated with stomatal and non-stomatal limitations (Chaves et al., 2009; Pan et al., 2020). Stomatal limitations are caused by stomatal closure, which reduces the amount of CO_2_ available for fixation. Salinity affects stomatal conductance immediately, first briefly through disturbed water relationships, then through local ABA synthesis. Stomatal conductance is also regulated by root signals such as ROS and Ca^2+^ waves (Fricke et al., 2004; Gilroy et al., 2014; Shabala et al., 2016).

The progressive sodium accumulation in photosynthetic tissues further inhibits CO_2_ assimilation, primarily affecting photosynthesis. This is called non-stomatal limitations of photosynthesis and is associated with the suppression of photosynthetic enzyme activity, the violation of chlorophyll biosynthesis, a decrease in the efficiency and structural integrity of the photosynthetic apparatus and thylakoid membranes, as well as an increase in non-photochemical quenching of chlorophyll fluorescence (Pan et al., 2020). The enlisted negative effects of non-stomatal limitations are associated with leaf ionic ion imbalances occurring primarily because of an excessive accumulation of Na^+^ and Cl^−^, K^+^ deficiency, and an increased level of reactive oxygen species (ROS) in the cytosol (Munns and Tester, 2008; Bose et al., 2014; Pan et al., 2020). An increase in Na^+^ concentration in the leaf apoplast was shown to inhibit PS II activity in intact mesophyll cells in a dose-dependent manner (Percey et al., 2014). A decrease in PS II activity correlates with the K^+^ efflux from cells, which indicates the requirement for K^+^ and Na^+^ homeostasis for the chloroplasts to function (Pottosin and Shabala, 2016). However, there is evidence that Na^+^ toxicity alone cannot fully explain the inhibitory effect of salinity on photosystem function (Kan et al., 2017; Pan et al., 2020). Overall, the effects of salinity on the primary processes of photosynthesis still remain largely understudied (Pan et al., 2020).

The key photosynthetic enzyme ribulose-1,5-bisphosphate carboxylase/oxygenase (Rubisco) is critical for regulating photosynthesis in the leaves of both C_3_ and C_4_ species. The response of Rubisco to abiotic stresses is not fully elucidated, as different studies give contradictory results. In glycophytes, a decrease in the content and activity of Rubisco under salinity is often observed (Lu et al., 2009; He et al., 2014). In halophytes, different reactions have been shown to depend on the salinity level; for example, a decrease in Rubisco activity at 100–250 mM NaCl and an increase at 400–500 mM NaCl in *Kalidium foliatum* (Gong et al., 2018). In *Haloxylon salicornicum*, an increase in Rubisco content occurred only at a sublethal concentration of NaCl (400 mM) (Panda et al., 2020). In the C_4_ species *Atriplex lentiformis* (in saline habitats), Rubisco activity remained constant per leaf area, but the activity of phosphoenolpyruvate carboxylase (PEPC)—the enzyme responsible for the initial CO_2_ fixation in the cytosol of mesophyll cells of C_4_ species—appeared to increase linearly along with increasing salinity (Zhu and Meinzer, 1999). The PEPC enzyme was revealed to be more salt sensitive *in vitro* than Rubisco, which appeared to be consistent with a lower salt concentration in the cytosol than in chloroplasts (Bose et al., 2017). Upregulation of PEPC in response to salinity or drought was detected in plants with C_4_ photosynthesis (Carmo-Silva et al., 2008; Cao et al., 2015).

Plant responses to low and high salinity vary, and the cellular and molecular mechanisms of salt-tolerance may also be significantly different (Shoukat et al., 2020; Zelm et al., 2020). The formation of an adaptive response to various salinities requires certain times. Studies on salt tolerant species (halophytes) have shown that both transport and the sequestration of sodium and potassium ions, as well as the synthesis and transport of compatible solutes, play a decisive role in salt tolerance mechanisms in plants (Volkov, 2015; Isayenkov and Maathuis, 2019; Zelm et al., 2020). This is largely due to the rate of sodium ions accumulating, which directly depends on the intensity and duration of salinization and the presence of specific mechanisms of salt tolerance.

*Kochia prostrata* (L.) Schrad. [*Bassia prostrata* (L.) A.J. Scott], forage kochia (subfamily Chenopodiaceae) is a widespread halophyte with C_4_ NADP type of photosynthesis, with a significant variety of morphological, biochemical, and ecological–physiological properties, high genetic polymorphism and a wide ecological plasticity (Shuyskaya et al., 2001; Gintzburger et al., 2003; Orlovsky et al., 2011; Akhzari et al., 2012; Toderich et al., 2013; Shuyskaya et al., 2020a). Forage kochia is a valuable forage plant for livestock grazing in the deserts and semi-deserts of central Eurasia (Harrison et al., 2000; ZoBell et al., 2003). *K. prostrata* is often referred to as the “alfalfa of the desert,” since it is comparable to alfalfa (*Medicago saliva* (L.)), and is esteemed for its ability to provide relatively large amounts of biomass, protein, carotene, phosphorous, and calcium to grazing animals in harsh, dry ecosystems (Davenport, 2005; Waldron et al., 2010). In winter months, forage kochia contains over 7.5% protein, having higher levels than 12 perennial grasses and three legumes (Koch, 2002; Davenport, 2005). It was found that *K. prostrata* had higher consumption as forage compared to C_3_ species *Artemisia herbaalba* and *Ceratoides lanata*, and C_4_ species *Atriplex canescens* (Nemati, 1977; McKell et al., 1989). In natural habitats, *K. prostrata* demonstrates great potential for establishing palatable perennial shrubs in arid rangeland with 70–220 mm of annual precipitation (Gintzburger et al., 2003; Bailey et al., 2010) and in saline soils (EC up to 20 dS/m) (Waldron et al., 2010). In model experiments, the decrease in biomass, water content, and accumulation of organic osmolytes following a 7–8-fold increase in sodium content and in the Na^+^/K^+^ ratio in *K. prostrata* leaves was shown under a long-term treatment of 200 mM NaCl (Karimi et al., 2005; Akhzari et al., 2012). However, at 50–150 mM NaCl treatment and 2–5 fold Na^+^ accumulation in leaves, the stability of growth parameters (dry biomass, relative growth rate, net assimilation rate), water use efficiency (WUE), as well as the content of water and organic osmolytes was observed. It was accompanied by a decrease in the content of chlorophyll and carotenoids as well as notable changes in the number and size of stomata (Karimi et al., 2005). It can be assumed that the slow accumulation of sodium ions leads to a specific adaptation of the photosynthetic apparatus that makes it possible to maintain the productivity of *K. prostrata* plants at low salinity (50–150 мМ NaCl). However, it is not clear how this adaptation occurs at the level of C_4_ enzymes of the carbon-concentrating mechanism (CCM) and the cyclic electron transport around photosystem I, which plays an important role in C_4_ plants. It is still unclear how long it may take to form an adaptive response to low (50–150 mM NaCl) and moderate (200 mM NaCl) salinity. Another question is whether the mechanisms of adaptation to 200 mM NaCl are the same as those to low salinity, or is a different mechanism triggered by the rapid accumulation of Na^+^ and its concentration approaching toxic levels? The study and determination of the duration and mechanisms of the formation of adaptive responses of halophytes to different salinity levels are necessary in order to improve approaches to increasing yields on saline soils (Zelm et al., 2020). We aimed to study the impact of 4- and 10-day treatments with low (100 mM NaCl) and moderate (200 mM NaCl) salinity on growth and photosynthetic processes to determine the duration and intensity of salinity required for the formation of an adaptive response of the C_4_ halophyte *K. prostrata*.

## Materials and methods

### Plant material and growth conditions

Seeds of *K. prostrata* (L.) Schrad. (forage kochia, *B. prostrata* (L.) A.J. Scott) were germinated on filter paper soaked in distilled water within 2–4 days. After that, the seedlings were transplanted to perlite in plastic pots of 24-cm length, 20-cm width, and 10-cm depth, with 20 seedlings per pot. Each plastic pot was placed on a separate plastic tray. During the next 30 days, the seedlings were grown using the nutrient solution 50% Hoagland, which was added to each plastic tray. The seedlings were grown in two separated climate chambers under circadian illumination (using commercial luminescent white light tubes): 8 h dark/16 h light (200 μmol/(m^2^ s) PAR, light meter LI-250A (Li-Cor, USA)) and 26 ± 2°C temperature. Experimental solutions of 100 mM NaCl and 200 mM NaCl were prepared on the basis of a 50% Hoagland solution. All solutions were added to a plastic tray. Plants were treated with NaCl for 4 and 10 days.

### Dry biomass and water content

At the end of the experiment, water content (W, g H_2_O g^−1^ DW) was assessed for the shoots in all the groups. Biomass was estimated for fresh (FW) and dry shoots (DW). Plant samples were dried at 80°C for 2 days until they reached a constant mass in order to measure quantitatively the dry shoot matter. The water content in the shoots for each treatment and control group was calculated as W = (FW − DW)/DW. Relative growth rate (RGR) between fourth and 10th days was calculated as RGR = (ln DW2 − ln DW1)/(t2 − t1), where DW1—dry weight at fourth day of treatment, DW2—dry weight at 10th day of treatment, t1— 4 days, and t2—10 days.

### Proline, Na^+^, and K^+^ ions contents

Free proline was determined according to Bates et al. (1973) with modifications. Dry shoot samples (0.2 g) from each group were homogenized in 2 ml of boiling distilled water, heated at 100°C for 10 min in a water bath, and then the homogenates were centrifuged (5 min, 14,000*g*). Approximately 1 ml of homogenate was reacted with 1 ml acidic ninhydrin (ninhydrin 1% (w/v) in glacial acetic acid 60% (v/v), 6 M orthophosphoric acid 40% (v/v)), and 1 ml glacial acetic acid in a tube for 1 h at 100°C in a water bath, and the reaction terminated in an ice bath. The mixtures were read at 520 nm using a Genesis 10 UV Scanning spectrophotometer (Thermo Scientific, USA). Proline concentrations were determined using a calibration curve and expressed as mmol g^−1^ DW. Na^+^ and K^+^ contents in the shoots were determined in water extracts from 100 mg dry samples on the flame photometer FPA-2-01 (AOOT ZOMZ, Russia), and expressed as mmol g^−1^ DW.

### CO_2_/H_2_O gas exchange

The CO_2_/H_2_O exchange was analyzed by placing a leaf segment into a temperature-controlled leaf chamber (26°C) where the sample was illuminated (1,200 µE PAR) through a fiber-optic light guide from a KL 1500LCD light source (Schott, Germany). The steady-state CO_2_/H_2_O exchange rates at the leaf–air interface were measured with a single-channel LI-820 infrared gas analyzer (LI-COR, USA) in the open-circuit mode. The leaf transpiration (*E*) was calculated from the difference in gas humidity at the inlet and outlet of the leaf chamber. In this experimental system, the humidity of gas flow at the entrance to the leaf chamber was kept constant at a known level using an LI-610 dew point generator (LI-COR). Humidity at the exit of the leaf chamber was determined with an HMP50 psychrometric sensor (Vaisala INTERCAP, Finland). Injected gas was atmospheric air preliminarily drawn into a 60-L polyethylene gasholder. A flow rate of 100 ml/min ensured continuous operation of the installation for 8 h. The mixing unit of the gas circuit made it possible to maintain a СО_2_ concentration of 400 ppm in the air flow supplied to the leaf chamber. After determining the photosynthetic CO_2_/H_2_O exchange, the light was switched off to measure the steady-state dark respiration (*Rd*) of leaves (Voronin, 2014). Water use efficiency (WUE, *A*/*E*) was calculated as the ratio of apparent photosynthetic assimilation (*A*) to the transpiration rate (*E*). *А*, *Е*, WUE, and *Rd* were measured at current (400 ppm) atmospheric concentration in the measuring chamber and low (200 ppm) СО_2_ concentration that existed in ancient times when evolution of the C_4_ lines occurred during 30 million years; this enabled us in the course of experiment to control stomatal opening, assess sensitivity of stomatal apparatus to external effects (Osborne and Sack, 2012; Taylor et al., 2018) and differentiate between stomatal and metabolic contributions to salt tolerance.

### Photosystem I

The redox potential changes of chlorophyll P700 were measured by monitoring the leaf absorbance at 820 nm using a dual-wavelength ED-P700DW pulse-modulated system (Walz, Germany) in combination with a PAM 101 fluorometer (Walz) (Klughammer and Schreiber, 1998). The kinetics of P700 oxidation were measured under illumination with far-red light (720 nm, 17.2 W m^−2^). The level of maximum P700 oxidation was determined by applying the flash from a xenon gas-discharge lamp (50 ms, 1,500 W m^−2^; Walz) in the presence of far-red light. The PS I cyclic electron transport activity was measured as the P700 oxidation kinetics in response to far-red illumination by monitoring changes in leaf absorbance (Nakamura et al., 2013).

### Photosystem II

The quantum yield of PS II photoreaction in dark adapted (20 min) leaves was determined with a pulse-amplitude-modulated chlorophyll fluorometer (PAM 101, Walz) (Schreiber, 1997). The ratio of variable to maximum chlorophyll *a* fluorescence (*F*_v_/*F*_m_) was used as a measure of the maximum quantum yield of the PS II reaction. During measurements, the sample was illuminated with weakly modulated red light. The output signal of PAM 101 was processed with an analog–digital convertor (PDA-100, Walz) and displayed on a computer. The potential photosynthetic efficiency of dark-adapted leaves was estimated from the values of minimal (*F*_0_) and maximal (*F*_m_) fluorescence using an expression: *F*_v_/*F*_m_ = (*F*_m_ − *F*_0_)/*F*_m_. Effective quantum yield (efficiency) of PS II photochemistry at a given light intensity was calculated as *Ф*_PSII_ = *F*_q_’/*F*_m_’, where *F*_q_’ is the photochemical quenching fluorescence by an open PS II reaction center and *F*_m_’ is the maximum fluorescence from a light-adapted leaf. Non-photochemical quenching of chlorophyll *a* fluorescence was calculated as NPQ = (*F*_m_ − *F*_m_’)/*F*_m_’.

### Protein extraction and Western blotting

Contents of ribulose-1.5-bisphophate carboxylase/oxygenase (Rubisco) and phosphoenolpyruvate carboxylas (PEPC) proteins were determined by means of immunoblotting analysis (Pozhidaeva, 2011). Total protein was extracted from 0.3 g of frozen plant shoots using a precooled mortar and pestle in 1 ml of ice-cold 50 mM Tris–HCl buffer (pH 8.2) containing 10 mM MgCl_2_, 0.3 mM EDTA, 40 mg of polyvinylpyrrolidone, and 5 mM dithiothreitol. Homogenate was centrifuged at 12 000 *g* for 15 min at 4°C. Protein content was assayed by the method of Bradford (1976) with bovine serum albumin (Sigma-Aldrich, USA) as a standard. A total of 20 μg of total protein samples were mixed with an equal volume of 50 mM Tris–HCl buffer (pH 8.2) containing 2% SDS, 10% glycerol, 100 mM DTT, and 0.1% bromophenol blue, denatured for 5 min at 70°C, and separated on 10% SDS-PAGE according to Laemmli (1970) using markers of standard molecular mass (BioRad, USA). After electrophoresis, proteins were transferred to a nitrocellulose membrane with a pore diameter of 0.45 μm (Amersham, GE Healthcare, UK) using a wet blotting system (BioRad, USA) according to the standard protocol. Transfer of proteins to the membrane was checked using 0.5% Ponceau S staining. Blots were blocked for 30 min at room temperature in buffer containing 5% low-fat milk in 1× PBS, 0.1% Tween-20 and hybridized with commercial polyclonal antibodies for proteins of large subunit (L) of Rubisco at a dilution of 1:10,000 (RbcL, AS03 037, Agrisera, Sweden), PEPC at a dilution of 1:5,000 (AS09 458, Agrisera, Sweden) for 1 h. Immunoreactions were detected using peroxidase-conjugated anti-rabbit IgG horse radish antibodies (Agrisera, AS09 602). The blots were developed with fluorescent dyes luminol and coumaric acid (Sigma, USA) in the presence of hydrogen peroxide and signals were visualized by Retina XBE film (Germany). The intensity of Western blotting bands was assessed using ImageJ 1.37v software (USA) and expressed relative to the average level (n = 3) for control plants, taken as 100%. The analysis was repeated at least three times.

### Antioxidant enzymes activity assay

Frozen plant shoots (0.3 g) were homogenized with a pre-chilled mortar and pestle in 0.1 M Tris–HCl (pH 7.4) containing 1 mM dithiothreitol (DTT), and 0.5 mM phenylmethylsulfonyl fluoride (PMSF) in DMSO. The homogenates were centrifuged at 10,000g for 15 min at 4°C. The supernatant was used for enzyme assays. Superoxide dismutase (SOD; EC 1.15.1.1) was determined with the 1.15 ml reaction mixture of 0.1 M Tris–HCl (pH 7.8), nitro blue tetrazolium (NBT, 50 μM), 10 mM L-methionine, 0.025% Triton X-100, 3 μM riboflavin, and 100 μl of enzyme extract based on the Beauchamp and Fridovich (1971) method. The absorbance was recorded at 560 nm after 2.5 min at the white light exposition (350 µmol m^−2^ s^−1^), and the SOD activity results were estimated as enzyme unit/mg protein. For detection of catalase (CAT; EC 1.11.1.6) activity, 100 μl of enzyme extract was combined with 2 ml of 0.1 M Tris–HCl (pH 7.4) and 0.5 ml of 0.1 M H_2_O_2_. Catalase activity was determined by a decrease in absorbance at 240 nm for 1 min due to H_2_O_2_ consumption (Aebi, 1984). The results were defined as µmol H_2_O_2_/(mg protein min).

### Statistical analyses

All of the physiological measurements were performed seven times. Factor (ANOVA) analyses were made using SigmaPlot 12.0 software. The figures show the means of the obtained values and their standard errors. Differences were considered significant at *P <*0.05 (Tukey’s test). Multiple factor principal component analysis (PCA) was conducted using R software (version 3.6.1).

## Results

### Biomass and RGR

**Figure 1:**
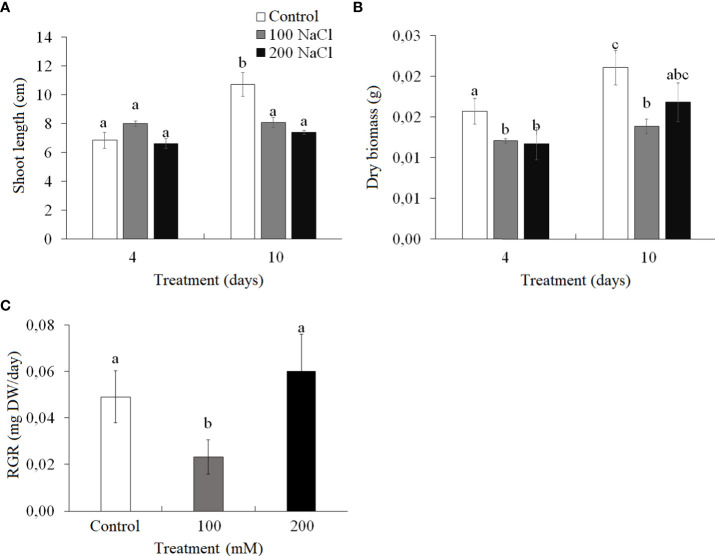
Effect of 4- and 10-day treatments with low (100 mM NaCl) and moderate (200 mM NaCl) salinity on plant biomass of *Kochia prostrata*. **(A)** Shoot length. **(B)** Dry biomass. **(C)** Relative growth rate (RGR) between fourth and 10th days NaCl treatment. Values are means ± standard errors of one experiment with three biological replicates. The different letters show statistically different means at *P* ≤0.05 (Tukey test).

On the fourth day of both 100 and 200 mM NaCl treatments, there were no significant changes in the length of *Kochia prostrata* shoots, while the dry weight of the shoots decreased by 23%–25% (**Figures 1A, B**). At the 10th day of 100 mM NaCl a low relative growth rate (RGR from the fourth to 10th days of treatment) was observed, while at 200 mM NaCl RGR remained the same as in control plants (**Figure 1C**).

### CO_2_/H_2_O gas exchange

**Figure 2:**
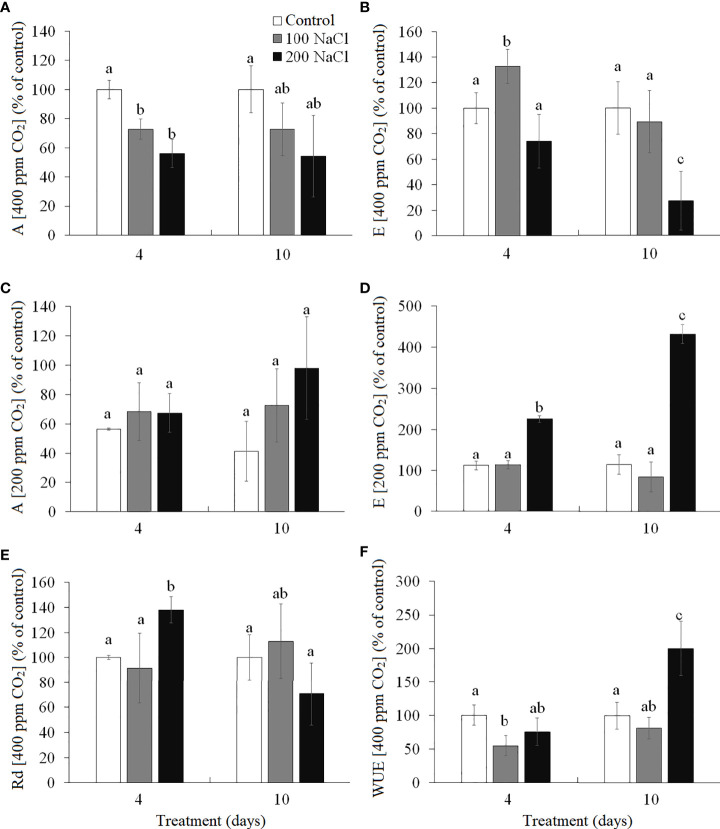
Effect of 4- and 10-day treatments with low (100 mM NaCl) and moderate (200 mM NaCl) salinity on CO_2_/H_2_O-gas exchange in *Kochia prostrata* shoots. **(A)** Apparent photosynthesis measured at 400 ppm CO_2_. **(B)** Transpiration intensity measured at 400 ppm CO_2_. **(C)** Apparent photosynthesis measured at 200 ppm CO_2_ (is expressed in percent of control at 400 ppm CO_2_). **(D)** Transpiration intensity measured at 200 ppm CO_2_ (is expressed in percent of control at 400 ppm CO_2_). **(E)** Dark respiration measured at 400 ppm CO_2_. **(F)** Water use efficiency (WUE) was calculated as the ratio of apparent photosynthesis to the transpiration rate (*A*/*E*) at 400 ppm CO_2_. Values are means ± standard errors of one experiment with three biological replicates. The different letters show statistically different means at *P* ≤0.05 (Tukey test).

The intensity of apparent photosynthesis (*A*, at 400 ppm CO_2_ concentration) decreased significantly by 30%–40% on the fourth day of both 100 and 200 mM NaCl treatments as compared with the control plants on the fourth day, but it did not differ significantly from the control plants on the 10th day (**Figure 2A**). Transpiration (*E*) at ambient CO_2_ (400 ppm) had increased by the fourth day of 100 mM NaCl treatment and decreased to the control level by the 10th day (**Figure 2B**). On the 10th day of treating plants with 200 mM NaCl, the transpiration was observed to have decreased by 30% as compared to the control plants. Measuring CO_2_/H_2_O gas exchange under conditions of low CO_2_ concentration (200 ppm) makes it possible to evaluate the stomatal limitations of photosynthesis. The intensity of apparent photosynthesis at 200 ppm CO_2_ decreased under all experimental conditions, while the expected increase in transpiration (associated with the artificial opening of stomata) took place only at 200 mM NaCl treatment: a 2.2-fold change was observed on the fourth day and a 4.3-fold one on the 10th day (**Figures 2C, D**. The change in the dark respiration intensity (*Rd*) at 400 ppm CO_2_ was observed on the fourth day of 200 mM NaCl treatment (38% increase). By the 10th day of both 100 mM NaCl and 200 mM NaCl treatments, *Rd* was at the level of the control plants (**Figure 2E**). Water use efficiency (*A*/*E*, 400 ppm CO_2_) significantly decreased (by 45%) by the fourth day of 100 mM NaCl treatment, while a 2-fold increase took place on the 10th day of 200 mM NaCl (**Figure 2F**).

### Photosystems I and II

**Figure 3:**
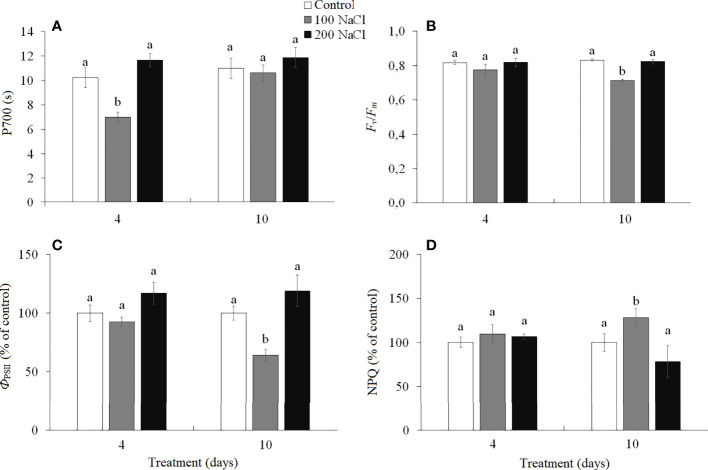
Effect of 4- and 10-days treatments with low (100 mM NaCl) and moderate (200 mM NaCl) salinity on photosynthetic parameters in *Kochia prostrata* shoots. **(A)** P700—time required to reach the maximum P700 oxidation level under far-red light (PSI); **(B)** *F_v_*/*F_m_*—maximum quantum yield of PSII; **(C)** *Ф*_PSII_—effective quantum yield of PSII at given light intensity; **(D)** NPQ—non-photochemical quenching of chlorophyll *a* fluorescence. Values are means ± standard errors of one experiment with three biological replicates. The different letters show statistically different means at *P* ≤0.05 (Tukey test).

Cyclic electron flow (CEF) around photosystem I (PS I) (measured as the time required to reach the maximum P700 oxidation level under far-red light) has been reduced by the fourth day of 100 NaCl treatment. Under all other treatments, the CEF remained at the level of the control plants (**Figure 3A**).

The maximum quantum yield of PS II (*F*_v_/*F*_m_) characterized the functioning of noncyclic electron flow in the photosynthetic ETC. On the 10th day of 100 mM NaCl treatment, a decrease (by 17%) in PS II efficiency was observed, associated with a decrease (by 35%) in the effective quantum yield (*Ф*_PSII_) and an increase (by 28%) in the intensity of non-photochemical quenching (NPQ) (**Figures 3B–D**). Under other treatments, the PS II efficiency remained at the level of the control plants (**Figures 3B–D**).

### Western blot analysis

**Figure 4:**
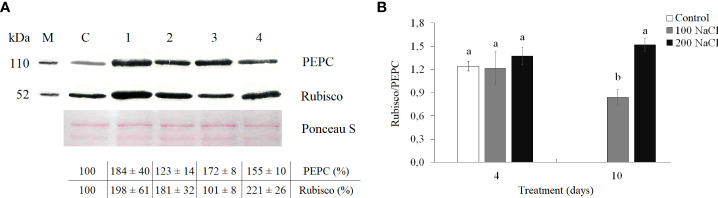
Effect of 4- and 10-day treatments with low (100 mM NaCl) and moderate (200 mM NaCl) salinity on Rubisco and PEPC contents in *Kochia prostrata* shoots. **(A)** Western blots for two key photosynthetic enzymes from total proteins extracted from shoots of *Kochia prostrata*. **(B)** Rubisco/PEPC ratio in *Kochia prostrata* shoots. Blots were probed with antibodies raised against Rubisco (subunit L) and PEPC. Numbers at the left indicate molecular mass in kilodaltons. M, marker; C, Control; 1, 4 days of 100 mM NaCl treatment; 2, 4 days of 200 mM NaCl treatment; 3, 10 days of 100 mM NaCl treatment; 4, 10 days of 200 mM NaCl treatment. Relative content of photosynthesis enzymes is shown on the basis of intensity of Western blotting bands estimated using ImageJ 1.37v software (United States) and expressed relative to average level for control plants taken as 100%. Equal protein loading was checked by staining of blots with Ponceau. Values are means ± standard errors of one experiment with three biological replicates. The different letters show statistically different means at *P* ≤0.05 (Tukey test).

The amount of Rubisco increased almost two-fold by the fourth day of both 100 and 200 mM NaCl. On the 10th day of 100 mM NaCl, Rubisco content decreased back to control values, but at 10 days of 200 mM NaCl, Rubisco content remained at the level of the 4-day treatment (**Figure 4A**, **Supplementary**). On days 4 and 10 of 100 mM NaCl, PEPC content was observed to be 1.7–1.8-fold higher as compared with those control plants. Whereas at 200 mM NaCl, a gradual increase in PEPC content was observed, by 1.2-fold at day 4 and by 1.5-fold at day 10 (**Figure 4A**, **Supplementary**). However, the Rubisco/PEPC ratio did not differ from the control values (1.2 ± 0.2) on the fourth day of both 100 and 200 mM NaCl, whereas, on the 10th day of 100 mM NaCl, the Rubisco/PEPC ratio decreased to 0.8 (**Figure 4B**).

### Water, proline, Na^+^, and K^+^ content, and SOD and CAT activity in shoots

**Figure 5:**
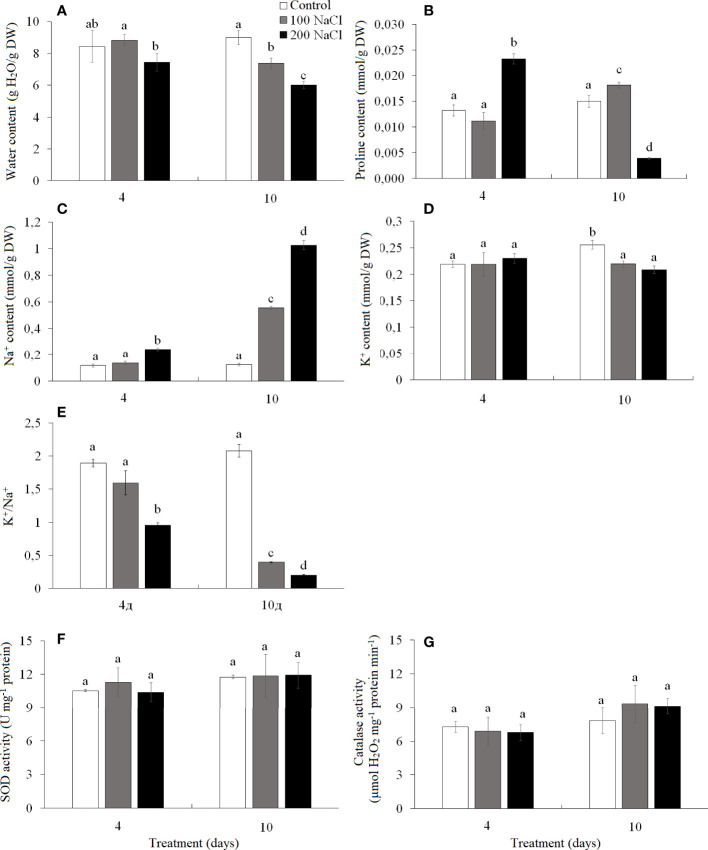
Effect of 4- and 10-day treatments with low (100 mM NaCl) and moderate (200 mM NaCl) salinity on the contents of water **(A)**, proline **(B)**, Na^+^ **(C)**, and K^+^ **(D)**, on the ratio of K^+^/Na^+^ **(E)** and on activities of catalase **(F)** and SOD **(G)** in *Kochia prostrata* shoots. Values are means ± standard errors of one experiment with three biological replicates. The different letters show statistically different means at *P* ≤0.05 (Tukey test).

The water content of *K. prostrata* shoots was significantly lower on the fourth day of 200 mM NaCl than on the fourth day of 100 mM NaCl. After a 10-day treatment, it decreased by 1.2 times at 100 mM NaCl and by 1.5 times at 200 mM NaCl as compared to the control plants (**Figure 5A**). The proline content on day 4 of 100 mM NaCl did not differ from the control level, while a 1.8-fold increase was observed on the fourth day of 200 mM NaCl treatment (**Figure 5B**). The proline content increased by 15% at day 10 of 100 mM NaCl and 4-fold decreased after a 10-day 200 mM NaCl treatment (**Figure 5B**).

Na^+^ accumulated 2-fold faster at 200 mM NaCl than at 100 mM NaCl. Thus, after 4 days of 100 mM NaCl treatment, Na^+^ content in *K. prostrata* shoots did not differ from the control plants, but at 200 mM NaCl it became two-fold higher. By the 10th day of treatment Na^+^ content was 4.5-fold higher at 100 mM NaCl, and 8.3-fold higher at 200 mM NaCl as compared to the control level (**Figure 5C**). After 4 days of 100 and 200 mM NaCl treatment K^+^ content did not differ from the control values and remained at the same level with further exposure to salinity, whereas control plants accumulated potassium 1.2-fold more by the 10th day of treatments (**Figure 5D**). K^+^/Na^+^ ratio decreased significantly under salinity; the change was especially significant at 200 mM NaCl treatment. Thus, in control plants K^+^/Na^+^ was 2.07 ± 0.01 and decreased to 1.6 and 1.0 by the fourth day of 100 and 200 mM NaCl, and 0.4 and 0.2 by the 10th day of 100 and 200 mM NaCl treatments, respectively (**Figure 5E**).

The activity of SOD and CAT did not change during 4–10 day periods under both 100 and 200 mM NaCl treatments (**Figures 5F, G**).

### Principal component analysis

**Figure 6:**
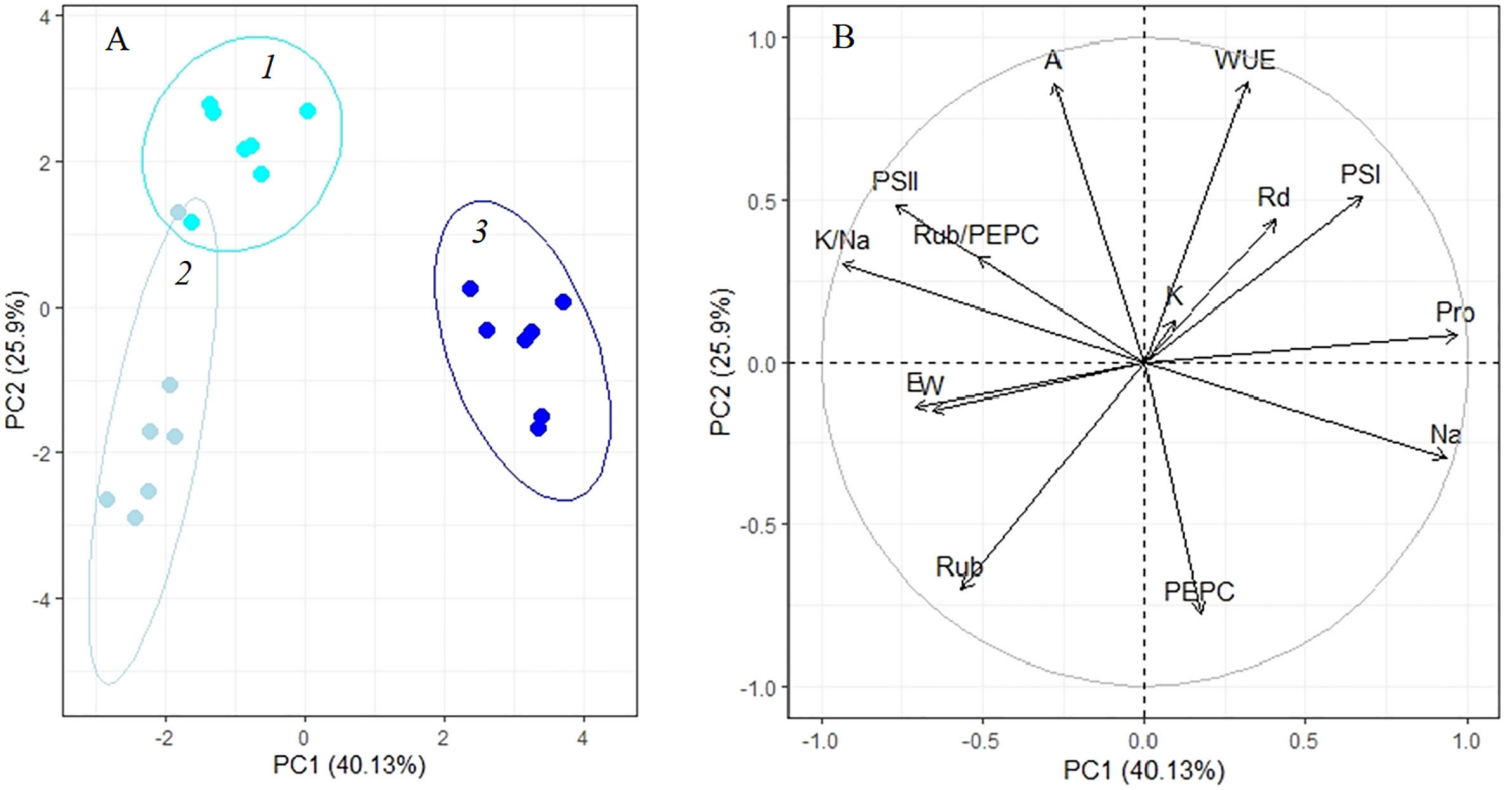
Principle component analysis (PCA) **(A)** score plot and **(B)** multiple correlation of the physiological data of *Kochia prostrata* on the fourth and 10th days of low (100 mM NaCl) salinity. *1*, Control; *2*, 4 days treatment; *3*, 10 days treatment. Parameters abbreviations are listed in **Table 1**.

Analysis of adaptation to a low (100 mM NaCl) salinity revealed that apparent photosynthesis, WUE, and key photosynthetic enzyme content (Rubisco and PEPC) played a major role in the 4-day treatment (PC2, **Figure 6A**, **Table 1**). Then, up to the 10th day of the procedure, the accumulation of osmolytes (sodium and proline) and the K^+/^Na^+^ ratio become especially significant (PC1, **Figure 6A**, **Table 1**). The first two principal components (PC1 and PC2) were enough to explain 66.03% of the pattern variation. There was a positive correlation between the accumulations of proline and sodium and a negative correlation between their contents and PS II efficiency and Rubisco/PEPC ratio (**Figure 6B**).

**Table 1:** Factor loading of morphophysiological and biochemical parameters on the main components (PC1 and PC2) of principal component analysis of C_4_ halophyte *Kochia prostrata* under 4- and 10-day treatments with low (100 mM NaCl) and moderate (200 mM NaCl) salinity.

Parameters	100 mM NaCl*		200 mM NaCl**		Total***	
	PC1	PC2	PC1	PC2	PC1	PC2
W	−0.2759	−0.0784	−0.2841	−0.0738	−**0.3257**	0.0707
Pro	**0.4089**	0.0433	−**0.3264**	0.2970	−**0.3361**	−0.0704
Na^+^	**0.3938**	−0.1557	**0.3593**	0.0984	**0.3607**	0.1428
K^+^	0.0388	0.0687	−0.1898	**0.5394**	−0.1645	−0.3701
K^+^/Na^+^	−**0.3932**	0.1598	−**0.3633**	−0.0410	−**0.3654**	−0.1713
PS I	0.2834	0.2675	0.1065	0.2446	0.1884	−0.3047
PS II	−0.3247	0.2552	0.0076	−0.0637	0.0926	−**0.4815**
Rub	−0.2379	−**0.3682**	0.2770	**0.3592**	0.1942	−0.0533
PEPC	0.0741	−**0.4082**	0.2886	0.1771	0.0812	**0.4795**
Rub/PEPC	−0.2171	0.1707	0.1544	0.2857	0.1357	−**0.4495**
A	−0.1177	**0.4526**	−0.2326	−**0.3501**	−0.2627	0.0646
Rd	0.1717	0.2300	−0.2689	0.3127	−0.2574	−0.1515
E	−0.2985	−0.0726	−**0.3045**	0.0026	−**0.3652**	0.0761
WUE	0.1344	**0.4543**	**0.3183**	−0.2788	**0.3389**	−0.1044

W, water content; Pro, proline content; PS I, PS II, photosystems I and II; Rub, Rubisco, ribulose-1.5-bisphophate carboxylase/oxygenase; PEPC, phosphoenolpyruvate carboxylase; A, apparent photosynthesis; Rd, dark respiration; E, transpiration; WUE, water use efficiency. The main significant factors are bold.

**Figure 7:**
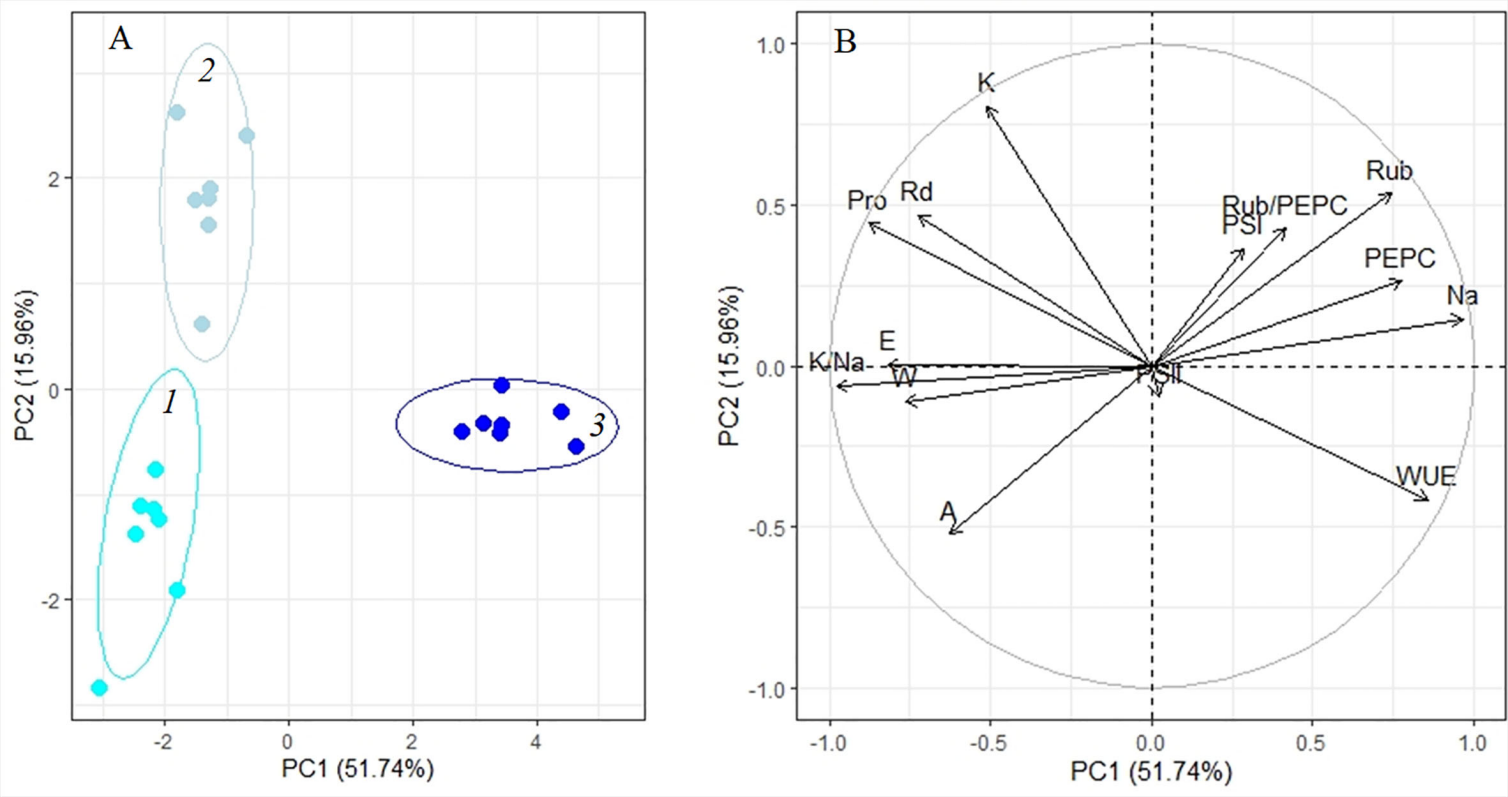
Principle component analysis (PCA) **(A)** score plot and **(B)** multiple correlation of the physiological data of *Kochia prostrata* on the fourth and 10th days of moderate (200 mM NaCl) salinity. *1*, Control; *2*, 4 days treatment; *3*, 10 days treatment. Parameters abbreviations are listed in **Table 1**.

**Figure 8:**
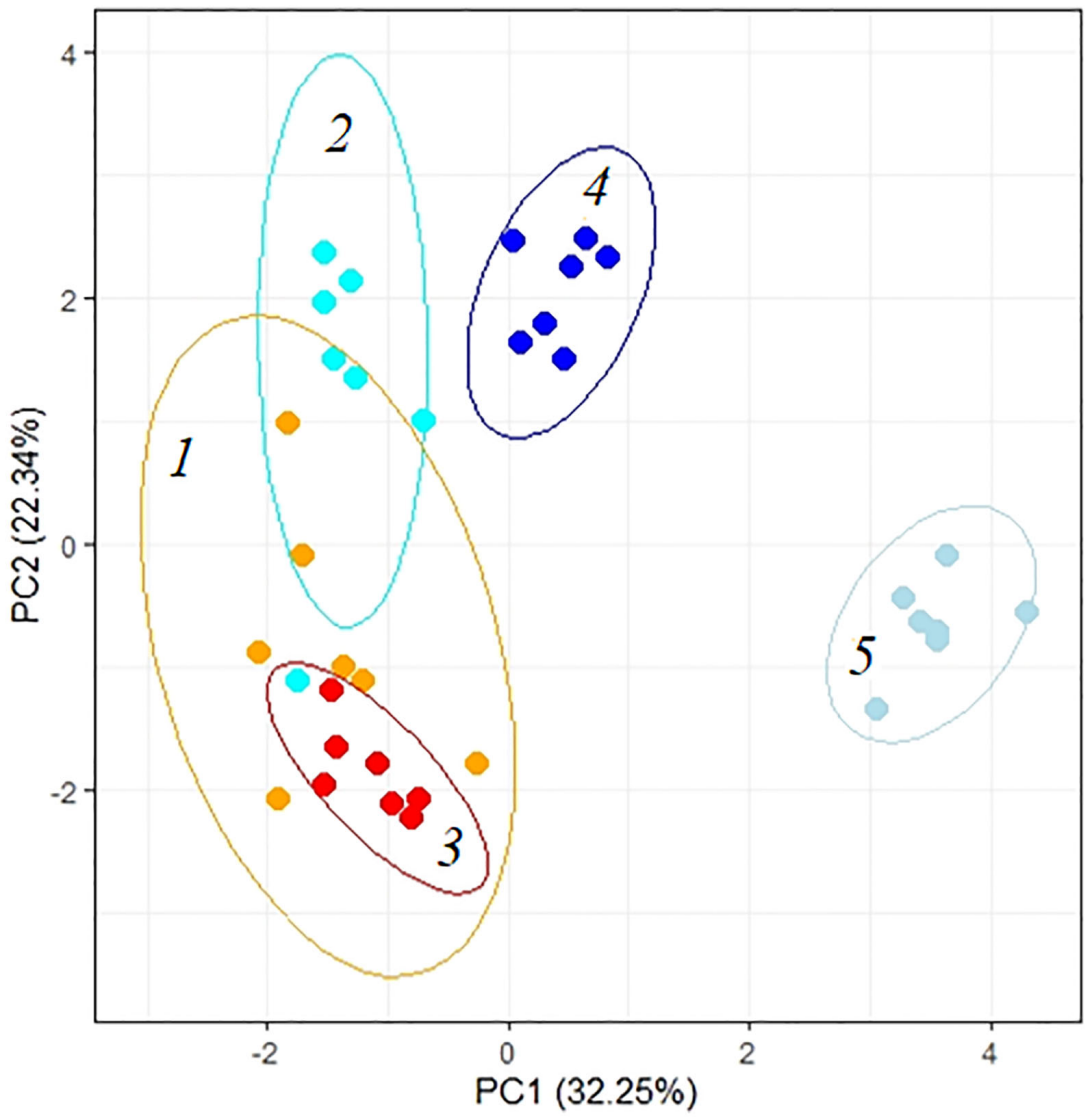
Principle component analysis (PCA) of the physiological data of *Kochia prostrata* on the fourth and 10th days of low (100 mM NaCl) and moderate (200 mM NaCl) salinity. *1*, Control; *2*, 4 days of 100 mM NaCl treatment; *3*, 4 days of 200 mM NaCl treatment; *4*, 10 days of 100 mM NaCl treatment; *5*, 10 days of 200 mM NaCl treatment. Parameters abbreviations are listed in **Table 1**.

*Factor loading of parameters on axes 1 and 2 of PCA on **Figure 6**, **Factor loading of parameters on axes 1 and 2 of PCA on **Figure 7**, ***Factor loading of parameters on axes 1 and 2 of PCA on **Figure 8**.

Analysis of adaptation to a moderate (200 mM NaCl) salinity showed that the potassium content, apparent photosynthesis, and Rubisco content played key roles at 4 days of treatment (PC2, **Figure 7A**, **Table 1**). After 10 days of salinity, the sodium and proline concentrations, K^+^/Na^+^ ratio, transpiration and WUE become more significant (PC1, **Figure 7A**, **Table 1**). The first two principal components (PC1 and PC2) were enough to explain 67.7% of the pattern variation. A positive correlation between the Na^+^ accumulation, Rubisco and PEPC contents, and WUE was observed (**Figure 7B**). However, under moderate salinity, the proline content appeared to negatively correlate with Na^+^ content (which is opposed to this correlation under low salinity). Negative correlation of the K^+^/Na^+^ ratio, water content and transpiration rate with the sodium accumulation was observed under both low and moderate salinity (**Figure 7B**).

Total PCA showed differences in the way *K. prostrata* plants respond to the duration and intensity of salinity. Significant factors in the division of *K. prostrata* reactions to 100 and 200 mM NaCl were associated with the efficiency of photosynthesis: PS II efficiency, content of the main C_4_ photosynthetic enzyme PEPC, and Rubisco/PEPC ratio (PC2, **Figure 8**, **Table 1**). Plants after 10 days of 200 mM NaCl treatment were clearly distinguished: a significant role was played by factors associated with the water-salt balance, namely, the main osmolyte (Na^+^) accumulation, changes in the K^+^/Na^+^ ratio, transpiration, WUE, water and proline contents (PC1, **Figure 8**, **Table 1**). The first two principal components (PC1 and PC2) were enough to explain 54.59% of the pattern variation.

## Discussion

### Response to 4–10 days of 100 мМ NaCl treatment

Salinity exposure inhibits plant growth, primarily due to the decrease in photosynthesis. Reduced CO_2_ assimilation rates may be caused by stomatal or non-stomatal limitations (Chaves et al., 2009; Pan et al., 2020). Absence of the effect of opening stomata at low CO_2_ (200 ppm) and decreased WUE indicate non-stomatal limitations of photosynthesis in *K. prostrata* after 4 days of 100 mM NaCl (**Figure 2**). However, the Na^+^ accumulation, changes in the K^+^/Na^+^ ratio and antioxidant enzymes activity in shoots were no observed under these conditions (**Figure 5**), although negative effects of non-stomatal limitations are usually considered as results of progressive Na^+^ accumulation, ionic ion imbalances in leaf, and an increase in the production of reactive oxygen species (ROS) (Pan et al., 2020). Proline accumulation in *K. prostrata* took place only after 10 days of salinity (by 1.8 times, **Figure 5B**), which is probably associated with sodium accumulation and osmotic potential regulation (**Figure 6B**).

Salt stress disrupts the delicate balance between photosynthetic electron transport and Calvin cycle reactions, which results in over-regeneration and an excess of energy within thylakoids. Excess energy can cause the overproduction of ROS and lead to photoinhibition of both photosystems (PS I and II) (Asada, 2006; Cerqueira et al., 2019). In *K. prostrata*, the decreased cyclic electron flow (CEF) around PS I was observed after 4 days of low salinity (**Figure 3A**). In C_4_ species, CO_2_ fixation requires more ATP than in C_3_ plants, since both the C_3_ and C_4_ cycles are functionally active there. Additional ATP is assumed to be produced by CEF due to the pH gradient being generated on the thylakoid membrane without NADPH being formed (Asada, 2000; Nakamura et al., 2013). In addition, CEF may play an important role in protecting photosystems from stromal hyperreduction-induced damage (Yamori et al., 2011; Yamori and Shikanai, 2016). It has been shown that CEF could take part in protecting the photosynthetic electron transport chain from the damage caused by drought in the maize (C_4_ species) (Zhou et al., 2019) and salinity (100 mM NaCl) in *Jatropha curcas* (CAM species) (Cerqueira et al., 2019). Perhaps, the decrease in CEF observed in *K. prostrata* under low salinity conditions is the result of the photosynthetic imbalance at the initial stage of salinity (4 days). After 10 days of salinity exposure CEF appeared to restore up to the control level. At the same time, a decrease in PS II efficiency took place at this stage due to photochemical quenching of chlorophyll fluorescence (NPQ) having increased (**Figure 3D**).

A 4-day 100mM NaCl treatment affected the content of photosynthetic enzymes Rubisco and PEPC in *K. prostrata* (**Figure 4A**). An increase in Rubisco content was observed in halophyte *Suaeda salsa* at 100–200 mM NaCl (Li et al., 2011), and in *K. foliatum* at 400–500 mM NaCl (Gong et al., 2018). At the same time, in C_4_ *A. lentiformis*, Rubisco activity remained constant, but PEPC activity increased in parallel with increasing salinity (Zhu and Meinzer, 1999). However, neither noticeable Na^+^ accumulation nor changes in the K^+^/Na^+^ ratio were observed in *K. prostrata* after 4 days of low salinity treatment (**Figure 5**). We assumed that an increase in Rubisco and PEPC content is associated with changes in CEF activity (**Figures 3A**, **4A**). After 10 days of salinity treatment, there was a significant decrease in the Rubisco/PEPC ratio (to 0.8) accompanied by an increase in CEF (**Figures 3A**, **4B**).

Thus, at low salinity levels, the decrease in biomass and significant changes in gas exchange parameters and photosynthetic enzyme content of *K. prostrata* were observed. However, after 4 days of salinity, the most significant reactions were a decrease in apparent photosynthesis and WUE, and an increase in Rubisco and PEPC contents (PC2, **Figure 6A**, **Table 1**), against the background of an unchanged level of Na^+^ and proline content in the shoots. By the 10th day of salinity, there was an accumulation of Na^+^ and proline in the shoots, which is necessary to stabilize PS I functioning and to reduce the Rubisco/PEPC ratio towards strengthening the C_4_ cycle. Plants grown at 50–100 mM NaCl salinity are considered an intermediate between osmotic stress and osmotic shock, when plants can cope with moderate adjustments in cell turgor and osmolarity without rapid activation of genes associated with osmotic and salt tolerance (Shavrukov, 2013). The stability of water and ion balance in *K. prostrata* reached by the fourth day of 100 mM NaCl treatment indicates the absence of any osmotic or salt shock. However, the changes observed in CEF PS I and the main photosynthetic enzyme content indicate an imbalance between photosynthetic electron transport and biochemical reactions that led to a decrease in the biomass and apparent photosynthesis in *K. prostrata.* The disturbances in photosynthetic electron transport may be caused by changes in cellular pH and ionic homeostasis, which have been triggered by early salinity signals (early sodium signaling wave) (Zelm et al., 2020). Changes in the content of key photosynthetic enzymes may be considered as a response to the restoration of the balance.

### Response to 4–10-days of 200 мМ NaCl treatment

The obtained data concerning the impact of moderate salinity on *K. prostrata* showed a decrease in biomass and intensity of apparent photosynthesis after 4 days of treatment and a recovery of RGR from the fourth to the 10th day of salinity (**Figures 1**, **2**). By the 10th day of 200 mM NaCl treatment at atmospheric CO_2_ concentration (400 ppm) the transpiration intensity has gradually decreased. At the same time, a 2–4-fold increase in transpiration was revealed to take place during the artificial opening of stomata (200 ppm CO_2_), which indicates the stomatal limitations of photosynthesis in *K. prostrata* (**Figure 2**). It is known that salinity immediately affects stomatal conductivity, induced by disturbed water relations and local ABA synthesis (Pan et al., 2020). The first phase of salinity normally exhibits osmotic stress, which takes place when the roots make contact with a saline substrate (Shavrukov, 2013). In *K. prostrata*, a gradual (1.5–2-fold) decrease in the transpiration intensity (**Figure 2B**), a decrease (20%–50%) in the water content (**Figure 5A**), and an increase (up to two times) in WUE (**Figure 2F**) may indicate the action of the osmotic component at 200 mM NaCl salinity. The increase in dark respiration after 4 days of moderate salinity (**Figure 2E**) may be considered as a dissipation of excess energy caused by a change in the ratio of CO_2_ assimilation to PS II efficiency, when CO_2_ assimilation decreased (**Figure 2A**), but photosystems functioned stably (**Figure 3**). In addition, the energy can be redirected to a twofold increase in proline synthesis (**Figure 5B**). It is known that P5CS1 (stress-specific isoform of proline) is localized in the cytosol and chloroplast (Szabados and Savoure, 2010; Kaur and Asthir, 2015). In chloroplasts, NADPH for P5CS1 synthesis comes from the photosynthetic electron transport chain (Liang et al., 2013; Shuyskaya et al., 2020b).

An increase in the content of photosynthetic enzymes (Rubisco and PEPC) in *K. prostrata* under moderate salinity was revealed to be associated with leaf Na^+^ accumulation (**Figure 7B**). In other words, an ion-specific effect is observed. A linear dependence between PEPC activity and the Na^+^ accumulation in leaves has been found in the C_4_ species *Kochia sieversiana* at 200 mM NaCl salinity (Ma et al., 2016). Such a fast increase in Rubisco content (as compared to that in PEPC) under salinity might be explained by Rubisco localization in bundle sheath cells in C_4_ species. These cells are adjacent to xylem and are characterized by a higher salt concentration than mesophyll cells, i.e., the cells that include PEPС (Bose et al., 2017). At 200 mM NaCl salinity, a rapid Na^+^ accumulation and a corresponding decrease in the K^+^/Na^+^ ratio were accompanied by a decrease in water content in *K. prostrata* (**Figures 5**, **7B**). Proline content doubled after 4 days of salinity (**Figure 5B**), which might be conditioned by its osmoregulatory function (Szabados and Savoure, 2010). After 10 days of moderate salinity, a sharp decrease in the proline content was observed (up to 6 times compared to the 4-day salinity) (**Figure 5B**). At the same time, the RGR of *K. prostrata* was restored to the control level (**Figure 1C**). Proline degradation is known to occur in mitochondria and provides the energy function of the oxidative pentose phosphate pathway (OPPP). Therefore, this process contributes to the energy supply for the recovery of plant growth after stress (Szabados and Savoure, 2010). In addition, since proline synthesis generates NADP^+^, and during proline oxidation NADPH is produced, the proline biosynthesis and degradation cycle is critical for redox buffering in various cellular organelles (Hare et al., 2003). Thus, at moderate salinity (200 mM NaCl), *K. prostrata* exhibits the reaction that is “typical” for salt-tolerant species. It was shown that in halophytes, salinity >100–150 mM NaCl rapidly activates many genes in response to osmotic shock and damage to the plasma membrane in root cells as well as to ion stress in shoot cells (Munns and Tester, 2008; Shavrukov, 2013). The present research showed that a 4-day salinity treatment results in a decrease in dry biomass and apparent photosynthesis. These consequences may be associated with an increase in sensitivity of stomata and in energy dissipation during dark respiration of *K. prostrata* (**Figures 1**, **2**). Active Na^+^ accumulation was accompanied by an elevated content of photosynthetic enzymes and WUE (**Figure 7B**). Multiple changes in the proline content and its positive correlation with Rd intensity (**Figures 5B**, **7B**) indicate that the proline actively takes part in metabolic flows in the cell. The total PCA clearly separated the 10-day treated plants with 200 mM NaCl from other experimental plant groups (**Figure 8**). It is the rapid Na^+^ accumulation, the active participation of proline, and an increase in WUE that contributed to the formation of a full-fledged adaptive response. During this CO_2_/H_2_O gas exchange, Rubisco and PEPC contents were stabilized due to stomatal regulation and led to biomass growth recovery back to control level.

In conclusion, the present study first revealed different types of response patterns of the C_4_ halophyte *K. prostrata* to low and moderate salinity on photosynthetic processes. New data were obtained on the imbalance between light and dark reactions of photosynthesis on the fourth day at low salinity (100 mM NaCl), which may indicate early salinity signaling in *K. prostrata*, in the absence of osmotic and ionic toxic stress (unchanged water and Na^+^ contents in shoots, increased transpiration, open stomata). In contrast, at moderate salinity (200 mM NaCl) in *K. prostrata*, there was a long-term effect of the osmotic component of salinity (stomatal limitations of photosynthesis, decreased water content in the shoots), and not the ionic one, despite a significant (4–8-fold) Na^+^ accumulation in the shoots. A decrease in dark respiration intensity, proline content in shoots, and an increase in water use efficiency and growth rate by the 10th day of 200 mM NaCl treatment point to the formation of a full-fledged adaptive response in *K. prostrata.* Our data on differences in responses to low and moderate salinity in *K. prostrata*, associated with the rate of sodium and proline accumulation in shoots, stomata sensitivity and dark respiration, the balance of light and dark reactions of photosynthesis, add new highlights to our understanding of the realization of different mechanisms of plant salt tolerance under various salinities.

## Data availability statement

The original contributions presented in the study are included in the article/**Supplementary Material**. Further inquiries can be directed to the corresponding author.

## Author contributions

ES, ZR, and MP designed and performed the experiments. ES, ZR, MP, LS, and PV performed the physiological and biochemical measurements. ES, ZR, MP, and KT analyzed the data and critically discussed the data. ES prepared the manuscript. ZR and KT reviewed and edited the manuscript. ES coordinated the research. All authors contributed to the article and approved the submitted version.

## Funding

The results were obtained within the state assignment of Ministry of Science and Higher Education of the Russian Federation (theme No. 122042700044-6) and also supported by the Bilateral Programs of Japan Society for the Promotion of Science (JSPS) and Science and Technology Research Partnership for Sustainable Development (SATREPS Project N 200101).

## Conflict of interest

The authors declare that the research was conducted in the absence of any commercial or financial relationships that could be construed as a potential conflict of interest.

## Publisher’s note

All claims expressed in this article are solely those of the authors and do not necessarily represent those of their affiliated organizations, or those of the publisher, the editors and the reviewers. Any product that may be evaluated in this article, or claim that may be made by its manufacturer, is not guaranteed or endorsed by the publisher.
