# Supplementary material for: Intensity and duration of salinity required to form adaptive response in C4 halophyte Kochia prostrata (L.) Shrad
Source: Front Plant Sci. 2022 Oct 7;13:955880. doi: 10.3389/fpls.2022.955880 (PMC9585317; doi:10.3389/fpls.2022.955880)
Supplement: Supplementary file 1 [file DataSheet_1.pdf]

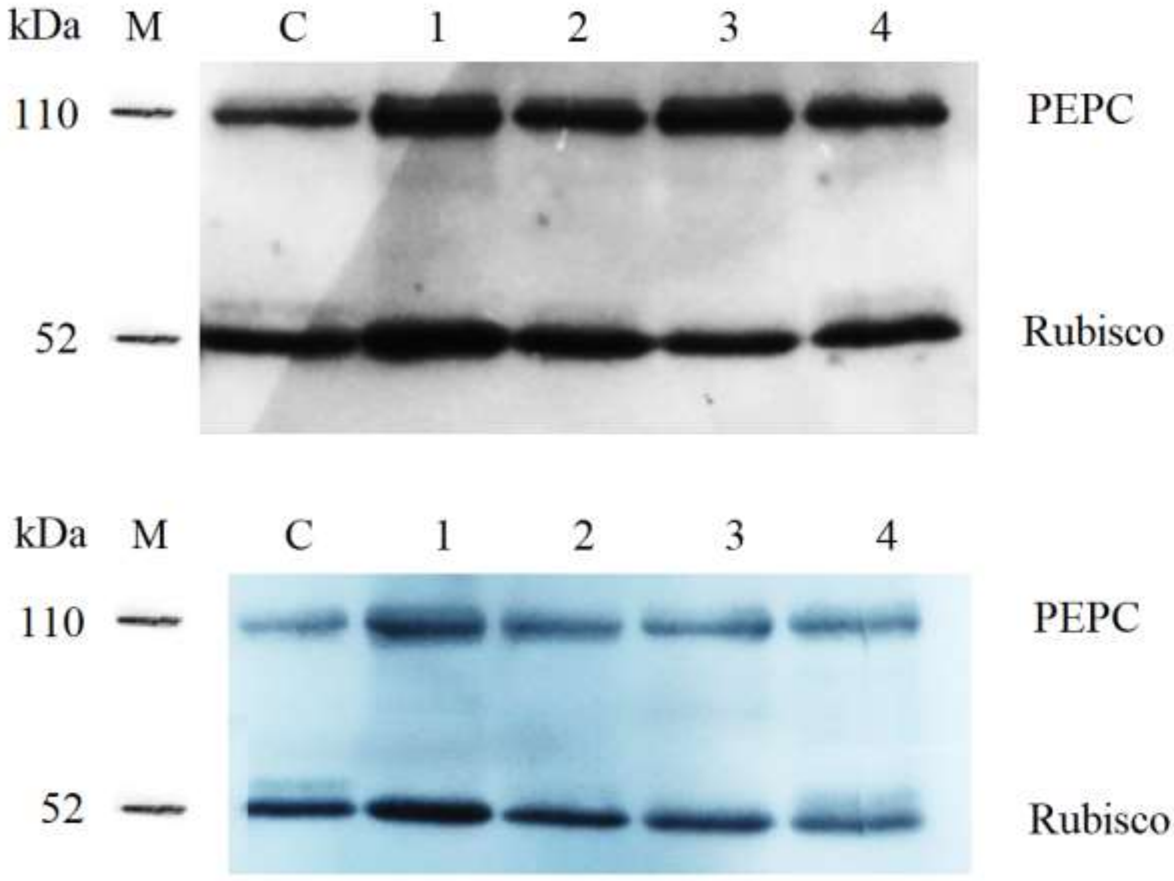

Supplementary Figure 1. The original images of Western blots for two key photosynthetic enzymes (Rubisco (subunit L) and PEPC) from total proteins extracted from shoots of *Kochia prostrata*. Numbers at the left indicate molecular mass in kilodaltons. M – marker, C – Control, 1 – 4 days of 100 mM NaCl treatment, 2 – 4 days of 200 mM NaCl treatment, 3 – 10 days of 100 mM NaCl treatment, 4 – 10 days of 200 mM NaCl treatment.
